# Case Report: Clinical and Hematological Characteristics of ε*γδβ* Thalassemia in an Italian Patient

**DOI:** 10.3389/fped.2022.839775

**Published:** 2022-03-17

**Authors:** Ilaria Fotzi, Francesco Pegoraro, Elena Chiocca, Tommaso Casini, Massimo Mogni, Marinella Veltroni, Claudio Favre

**Affiliations:** ^1^Department of Pediatric Hematology/Oncology and Hematopoietic Stem Cell Transplantation (HSCT), Meyer Children's University Hospital, Florence, Italy; ^2^Department of Health Science, University of Florence, Florence, Italy; ^3^Human Genetics Laboratory, Istituto di Ricovero e Cura a Carattere Scientifico (IRCCS) Istituto Giannina Gaslini, Genoa, Italy

**Keywords:** thalassemia, ε*γδβ*, children, newborn, anemia

## Abstract

**Introduction:**

ε*γδβ* thalassemia is a rare form of β-thalassemia mostly described in children originating from Northern Europe. Only anecdotic cases from the Mediterranean area are reported. The diagnosis is challenging, considering the rarity of the disease and its heterogeneous clinical presentation. Most patients have neonatal microcytic anemia, sometimes requiring in *utero* and/or neonatal transfusions, and typically improving with age.

**Case Description:**

We report on an Italian newborn presenting with severe neonatal anemia that required red blood cell transfusion. After the first months of life, hemoglobin levels improved with residual very low mean corpuscular volume. β and α thalassemia, IRIDA syndrome, and sideroblastic anemia were excluded. Finally, a diagnosis of ε*γδβ* thalassemia was made after microarray analysis of single nucleotide polymorphisms revealed a 26 kb single copy loss of chromosome 11p15.4, including the *HBD, HBBP1, HBG1, and HBB* genes.

**Conclusions:**

Despite its rarity, the diagnosis of ε*γδβ* thalassemia should be considered in newborns with severe neonatal anemia requiring in *utero* and/or neonatal transfusions, but also in older infants with microcytic anemia, after excluding more prevalent red blood cell disorders.

## Introduction

The ε*γδβ* thalassemia is an extremely rare heterozygous form of β-thalassemia, with around 40 reported cases in 2019 (1). In most cases, patients originated from ethnic backgrounds where β-thalassemia was not prevalent (Table 1). Despite the extreme heterogeneity of the molecular bases of β-thalassemia in Italy, the first ε*γδβ* thalassemia deletion has been only identified in 2016 (23).

**Table 1:** Origin and presentation of previously described patients with ε*γδβ* thalassemia.

**Ethnic origin**	**Intrauterine presentation**	**Neonatal**		**Neonatal transfusion**	**Outcome at last follow-up**	**References**
		**Hb (g/dL)**	**MCV (fL)**			
Anglo-Saxon	n.r.	10.4	84	+	Mild Anemia	(2)
Dutch	Still birth	n.r.	n.r.	+	Died after birth	(3)
Mexican American	n.r.	n.r.	n.r.	+	Mild Anemia	(4)
English	n.r.	n.r.	n.r.	–	Mild Anemia	(5)
Croatian	n.r.	n.r.	n.r.	–	Mild Anemia	(6)
Canadian	n.r.	n.r.	n.r.	+	Mild Anemia	(6)
Hispanic	n.r.	n.r.	n.r.	+	Mild Anemia	(7)
Scottish Irish	n.r.	n.r.	n.r.	+	Mild Anemia	(8)
Scottish Irish	n.r.	n.r.	n.r.	+	Mild Anemia	(8)
Scottish Irish	Intrauterine transfusion	12.2	83	+	Mild Anemia	(8)
Irish	n.r.	n.r.	n.r.	+	Mild Anemia	(9)
Dutch	n.r.	n.r.	n.r.	–	Mild Anemia	(10)
Chilean	Intrauterine transfusion	10.2	51	+	Mild Anemia	(11)
Dutch	n.r.	7.9*	92*	+	Mild Anemia	(12)
English	n.r.	n.r.	n.r.	+	Mild Anemia	(13)
English	n.r.	n.r.	n.r.	+	Mild Anemia	(13)
English	n.r.	n.r.	n.r.	+	Mild Anemia	(13)
Japanese	n.r.	n.r.	n.r.	–	Mild Anemia	(14)
Norwegian	Intrauterine transfusion	n.r.	n.r.	+	Mild Anemia	(15)
French	Intrauterine transfusion	n.r.	n.r.	+	Mild Anemia	(16)
Irish Scottish	n.r.	8.6	90	+	Mild Anemia	(17)
Irish Scottish	Intrauterine transfusion	10.3*	86*	+	Mild Anemia	(17)
English	n.r.	n.r.	n.r.	–	Mild Anemia	(18)
Swiss	Pathologic cardiotocography	n.r.	n.r.	+	Hypochromic anemia	(19)
Bedouin	n.r.	n.r.	n.r.	–	Died immediately	(20)
Bedouin	n.r.	6	n.r.	+	Mild Anemia	(20)
Bedouin	n.r.	6.2	n.r.	+	Mild Anemia	(20)
Bedouin	n.r.	n.r.	n.r.	–	Stillborn	(20)
Bedouin	n.r.	10	n.r.	+	Mild Anemia	(20)
Bedouin	n.r.	5.6	n.r.	+	Mild Anemia	(20)
Bedouin	n.r.	10	n.r.	–	Birth Asphyxia; dead	(20)
Bedouin	n.r.	5	n.r.	+	Brain Hypoxia	(20)
Bedouin	n.r.	8.5	n.r.	+	Mild Anemia	(20)
Bedouin	n.r.	8.6	n.r.	+	Mild Anemia	(20)
Austrian	n.r.	n.r.	n.r.	–	Mild Anemia	(21)
English	n.r.	n.r.	n.r.	–	Mild Anemia	(22)
English	Intrauterine transfusion	n.r.	n.r.	+	Mild Anemia	(22)
English	n.r.	n.r.	n.r.	–	Mild Anemia	(22)
English	n.r.	n.r.	n.r.	–	Mild Anemia	(22)
English	n.r.	n.r.	n.r.	+	Mild Anemia	(22)
Italian	n.r.	n.r.	n.r.	–	Mild Anemia	(23)
North American	Intrauterine transfusion	n.r.	n.r.	+	n.r.	(24)
Chinese	Intrauterine transfusion	12.8	n.r.	+	Mild Anemia	(25)
Spanish	n.r.	6.4	87.1	+	Mild Anemia	(26)
European Caucasian	n.r.	6.6	69.4	+	Mild Anemia	(1)
European Caucasian	n.r.	8	88	+	Mild Anemia	(1)
Italian	n.r.	10.8	65.4	+	Microcytosis	Our report

n.r., not reported; *post transfusion; +, required; -, not required.

ε*γδβ* thalassemias are caused by long deletions in the β-globin cluster and exist only in heterozygous form. Except for one case (8, 27), the reported deletions are almost exclusively unique and in most cases *de novo*, explaining the phenotypic heterogeneity of the disease. Indeed, multiple clinical phenotypes of ε*γδβ* thalassemia have been reported, ranging from normal blood cell count to severe anemia requiring in *utero* and/or neonatal transfusions (Table 1) (20). The underlying reasons for such a spectrum of clinical characteristics are unknown, but the type and length of the deletion are not responsible, as contrasting phenotypes have been reported in heterozygotes with identical deletions within the same family (8). At the molecular level ε*γδβ* thalassemias fall into two distinct categories: in group I all, or a greater part of the β-globin cluster, are removed, including the β-globin gene, whereas in group II extensive upstream regions are removed, leaving the β-globin gene itself intact although its expression is silenced because of inactivation of the upstream β-locus control region (23). Furthermore, co-existent α-globin gene triplication has been suggested to exacerbate the phenotype of ε*γδβ* thalassemia increasing the imbalance between the α and non-α globin chain ratio during fetal life (16).

Most patients with ε*γδβ*-thalassemia had neonatal erythroblastosis, reticulocytosis, hypochromia, and microcytosis (Table 1), that later improved with age. Anemia usually remitted spontaneously during the first months of life, and the adult phenotype is similar to that of the β-thalassemia trait, but with more severe microcytosis (13).

Herein, we describe the clinical phenotype of a novel Italian ε*γδβ* deletion, the second patient from Italy described in the literature and the third from the Mediterranean Area, presenting with severe microcytic anemia in the neonatal period.

## Case Description

**Figure 1:**
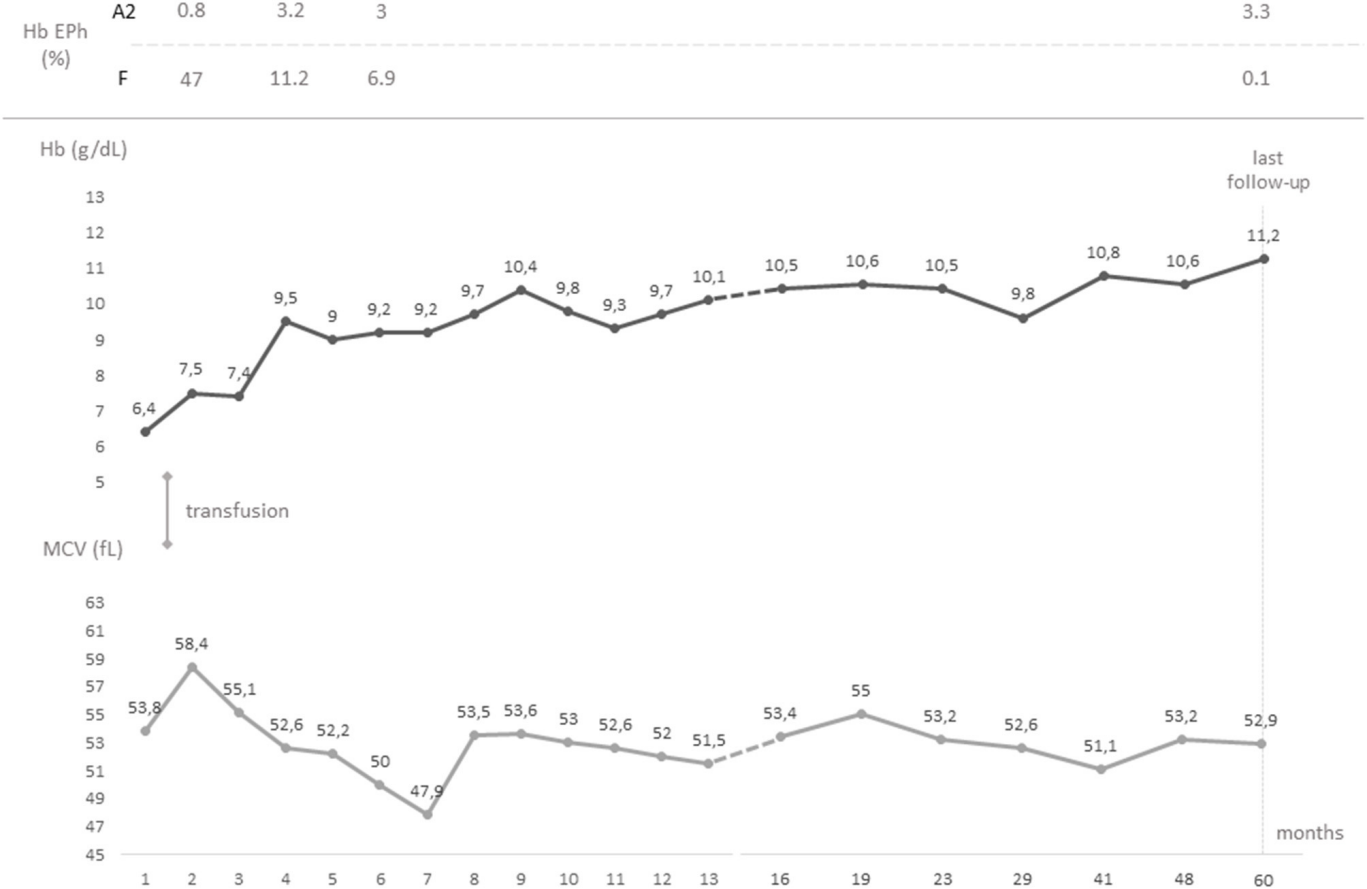
Timeline graph showing the chronological evolution of the values of hemoglobin and mean corpuscular volume from birth to last follow-up. Hemoglobin electrophorese studies and transfusions are also reported. Hb, hemoglobin; EPh, electrophoresis; MCV, mean corpuscular volume.

**Figure 2:**
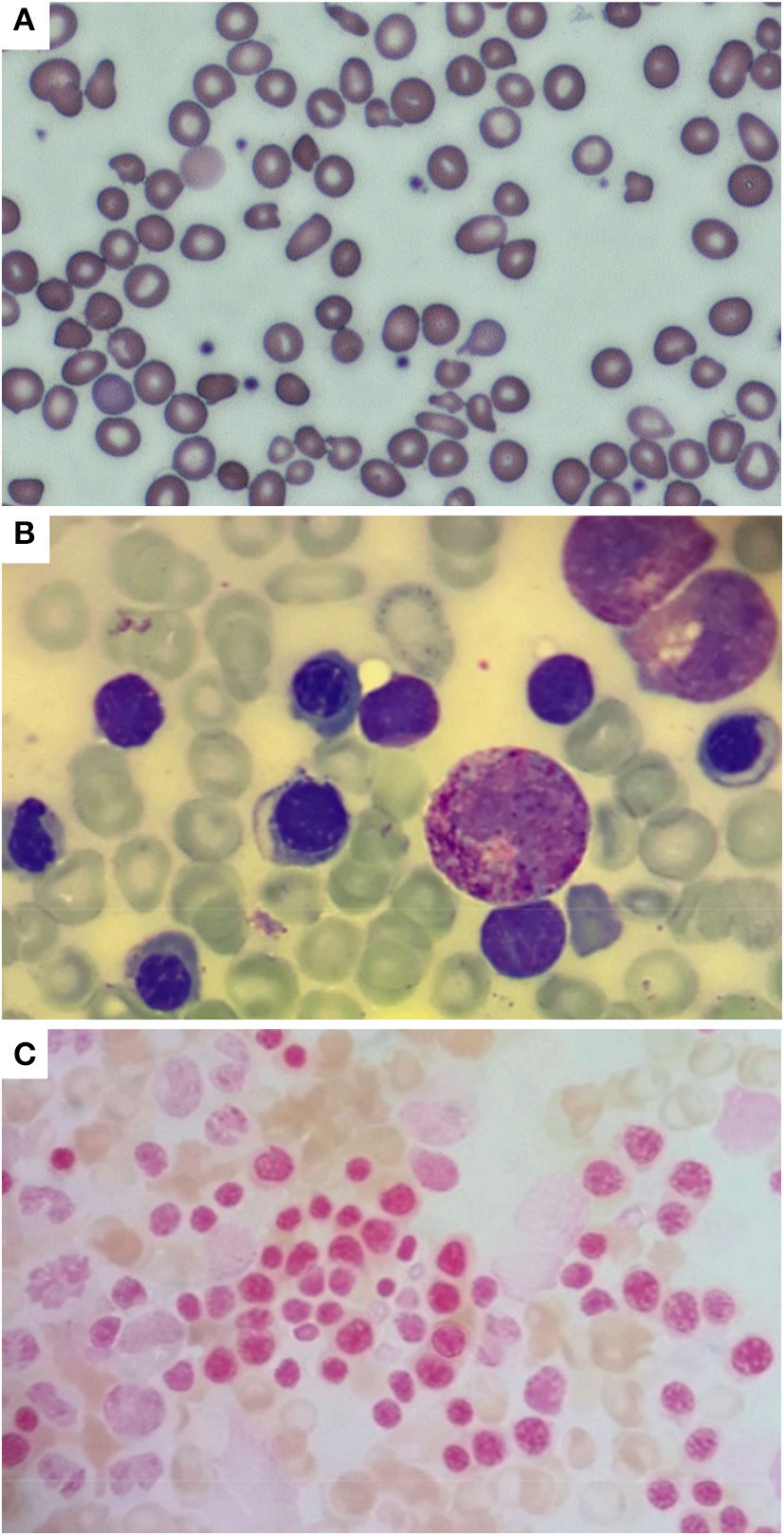
Peripheral blood smear **(A)** performed in the neonatal period, showing hypochromic erythrocytes with anisopoikilocytosis; isolated target cells, ovalocytes, ellissocytes, and dacrocytes are also visible (600x magnification, MGG). Bone marrow aspirate **(B)** performed at 2 months of age showing mild dyserythropoiesis (1000x magnification, MGG); no ring sideroblasts were found in the smear [**(C)** 1000x magnification, Pearls coloration].

A male, full-term infant of Tuscanian origin was born by induced vaginal delivery due to meconium-stained amniotic fluid. He presented with clinical and laboratory signs of sepsis (increased white blood cell count, C-reactive protein, and indirect bilirubin) and received wide spectrum antibiotics. Laboratory evaluations revealed microcytic anemia (hemoglobin, Hb, 10.8 g/dL, mean corpuscular volume, MCV, 65.4 fL). The clinical condition rapidly improved and hemoglobin rose to 12 g/dL, with persistent microcytemia. At the first follow-up visit at 1 month of age, hemoglobin had dropped to 6.4 g/dL, with a MCV of 53.8 fL, a mean cell hemoglobin concentration of 18.3 g/dL, a hematocrit of 19.9%, and an increased reticulocyte count (0.2 × 10^6^/L) (Figure 1). No other signs of hemolysis were detected (normal bilirubin and lactate dehydrogenase levels). The peripheral blood smear revealed microcytic hypochromic erythrocytes with anisopoikilocytosis (Figure 2). Hemoglobin electrophoresis showed a normal pattern with an unusually high proportion of HbA (HbF 47%, HbA2 0.8%, HbA 52.2%), no abnormal hemoglobin variants, nor evidence of β-thalassemia; abdominal ultrasound showed splenomegaly. The patient received a red blood cell transfusion and supplementation of iron and folic acid, which proved ineffective. Therefore, bone marrow aspiration was performed to exclude the presence of ring sideroblasts with Prussian blue staining (Figure 2); normal plasmatic hepcidin values ruled out an Iron-Refractory Iron Deficiency Anemia (IRIDA) syndrome.

**Figure 3:**
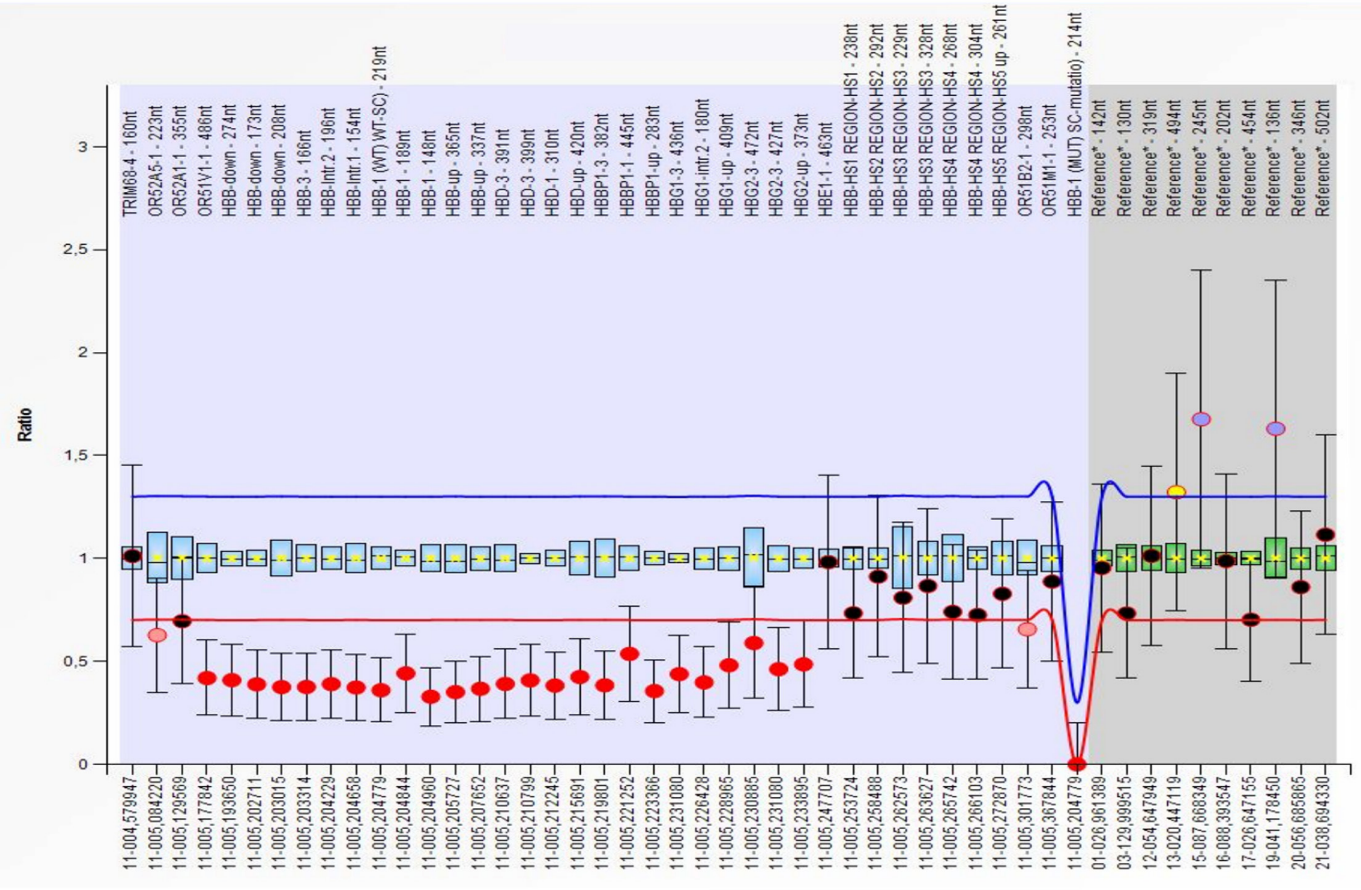
Multiplex Ligation Probe Amplification (MLPA) showing a deletion of the *OR51V1-1, HBB, HBD, HBBP1, HBG1* and *HBG2* genes (red dots) on the short arm of chromosome 11. All deletion were detected in the heterozygous form. The first deleted probe was the 486 on the *OR51V1-1* gene (hg18 loc.11–005,177842), while the last was the 373, after the end of the *HBG2* gene (hg18 loc.11–005,233895).

At 6 months of age, the blood cell count of the patient was consistent with a thalassemia trait (hemoglobin 9 g/dL, red blood cell 6.19 × 10^12^/L, MCV 52 fL, mean cell hemoglobin, MCH, 6.3 pg). The hemoglobin electrophoresis showed HbA2 value of 3.3%, and α gene deletions were excluded using Multiplex Ligation Probe Amplification (MLPA). Conversely, MLPA showed a heterozygous deletion in the short arm of chromosome 11 (Figure 3). This was confirmed by microarray analysis of single nucleotide polymorphisms that revealed a 26 kb single-copy loss of a genomic region localized at 11p15.4. The lost genetic material included the *HBD, HBBP1, HBG1*- and partially *HBB*- genes, a finding consistent with ε*γδβ* thalassemia. The family history was negative for similarly affected individuals and targeted parental testing via quantitative polymerase chain reaction confirmed the presence of a *de novo* deletion.

At last follow-up (5 years of age), the patient had a hemoglobin of 11.2 g/dL, a MCV of 52.9 fL, and a MCH of 17 pg; hemoglobin electrophoresis revealed 0.1% of HbF and 3.3% of HbA2 (Figure 1). The patient was in good clinical condition, with normal growth (72^nd^ centile of height and 80^th^ centile of weight, WHO curves) and cognitive development. No splenomegaly was found at the abdominal ultrasound, nor signs of iron overload/deficiency. Therefore, no specific follow-up plan nor specific interventions in case of minor ailments were deemed necessary, as for β-trait carriers.

## Discussion

Anemia during the neonatal period represents a challenge for the pediatrician, mainly for the multiplicity of conditions that are responsible for the condition during the first weeks of life. The etiology of neonatal anemia usually falls into three major categories: blood loss, decreased production, and increased destruction of erythrocytes (28). The differential diagnosis for hemolytic anemia in the newborn period includes alloimmunity, erythrocyte membrane defects, enzyme deficiencies, and hemoglobinopathies. The most frequent hemoglobinopathy associated with critically ill infants and hemolytic anemia is α thalassemia with deletion of three α globin genes (28, 29).

ε*γδβ* thalassemia usually presents as severe neonatal hemolytic anemia that requires in *utero* and/or neonatal transfusions but this condition is rarely considered among the causes of neonatal anemia and therefore misdiagnosed, as in our case. A reduced MCV without abnormalities on hemoglobin electrophoresis in a newborn is not always detected in ε*γδβ* thalassemia (Table 1), but when it is found, it can orient toward the diagnosis. Despite the high incidence of thalassemias in Italy, the significant microcytosis in our patient was initially deemed secondary to iron deficiency, as the intercurrent sepsis misdirected high indirect bilirubin values as a sign of hemolysis.

Although uncommon during the neonatal period, microcytosis can occur secondary to iron deficiency following feto-maternal hemorrhage. However, in most cases, it is associated with thalassemia, also depending on the α thalassemia allele frequency, which varies in different populations (30). After the neonatal period, the hematologic phenotype of microcytosis associated with normal hemoglobin electrophoresis, which is typical of ε*γδβ* thalassemia, can be associated to or confused with α thalassemia, but also, in presence of normal ferritin levels, with IRIDA. Unlike previously suggested, the severe phenotype of our patient was not justified by the presence of α triplication, which was excluded by MLPA analysis.

There is no established explanation for the phenotypic heterogeneity of the disease, but it is not dependent on the type and length of deletion (8). Although at the molecular level ε*γδβ* thalassemias fall into two distinct categories, the associated phenotypes of the two groups are similar. Therefore, the variable severity is likely to be influenced by other genetic and environmental factors.

The remission of anemia after the first months of life is a consequence of the increasing production of β-globin that reduces the imbalance between α/non-α globin chain synthesis. The residual adult phenotype is similar to that of the β-thalassemia trait but with normal, rather than increased, levels of hemoglobin A2 due to the loss of one δ locus, while the fetal hemoglobin is normal or minimally increased (13). The normal HbA2 levels make the hematologic phenotype also similar to that of carriers of α-thalassemia (23). However, data collected by Rooks et al. suggest that adult heterozygotes for ε*γδβ*-thalassemias tend to have more severe microcytosis and hypochromia even than β^0^-thalassemia carriers (13).

In conclusion, this case remarks the importance of considering the ε*γδβ* thalassemia in the differential diagnosis of hypochromic microcytic hemolytic anemias in the newborn period. In the post-natal period, microcytosis with normal ferritin values and without abnormalities on hemoglobin electrophoresis should also raise the suspicion for ε*γδβ* thalassemia.

## Data Availability Statement

The original contributions presented in the study are included in the article/supplementary material, further inquiries can be directed to the corresponding author.

## Ethics Statement

Ethical review and approval was not required for the study on human participants in accordance with the local legislation and institutional requirements. Written informed consent to participate in this study was provided by the participants' legal guardian/next of kin. Written informed consent was obtained from the minor(s)' legal guardian/next of kin for the publication of any potentially identifiable images or data included in this article.

## Author Contributions

IF and FP wrote the manuscript and MV and CF critically reviewed it. IF, EC, and TC followed the patient. MM performed the genetic analysis. All authors contributed to the article and approved the submitted version.

## Conflict of Interest

The authors declare that the research was conducted in the absence of any commercial or financial relationships that could be construed as a potential conflict of interest.

## Publisher's Note

All claims expressed in this article are solely those of the authors and do not necessarily represent those of their affiliated organizations, or those of the publisher, the editors and the reviewers. Any product that may be evaluated in this article, or claim that may be made by its manufacturer, is not guaranteed or endorsed by the publisher.
